# Phenotypic Responses of Differentiated Asthmatic Human Airway Epithelial Cultures to Rhinovirus

**DOI:** 10.1371/journal.pone.0118286

**Published:** 2015-02-23

**Authors:** Jianwu Bai, Steven L. Smock, George R. Jackson, Kenzie D. MacIsaac, Yongsheng Huang, Courtney Mankus, Jonathan Oldach, Brian Roberts, Yu-Lu Ma, Joel A. Klappenbach, Michael A. Crackower, Stephen E. Alves, Patrick J. Hayden

**Affiliations:** 1 Merck Research Laboratories, Boston, Massachusetts, United States of America; 2 MatTek Corporation, Ashland, Massachusetts, United States of America

## Abstract

**Objectives:**

Human airway epithelial cells are the principal target of human rhinovirus (HRV), a common cold pathogen that triggers the majority of asthma exacerbations. The objectives of this study were 1) to evaluate an *in vitro* air liquid interface cultured human airway epithelial cell model for HRV infection, and 2) to identify gene expression patterns associated with asthma intrinsically and/or after HRV infection using this model.

**Methods:**

Air-liquid interface (ALI) human airway epithelial cell cultures were prepared from 6 asthmatic and 6 non-asthmatic donors. The effects of rhinovirus RV-A16 on ALI cultures were compared. Genome-wide gene expression changes in ALI cultures following HRV infection at 24 hours post exposure were further analyzed using RNA-seq technology. Cellular gene expression and cytokine/chemokine secretion were further evaluated by qPCR and a Luminex-based protein assay, respectively.

**Main Results:**

ALI cultures were readily infected by HRV. RNA-seq analysis of HRV infected ALI cultures identified sets of genes associated with asthma specific viral responses. These genes are related to inflammatory pathways, epithelial structure and remodeling and cilium assembly and function, including those described previously (e.g. CCL5, CXCL10 and CX3CL1, MUC5AC, CDHR3), and novel ones that were identified for the first time in this study (e.g. CCRL1).

**Conclusions:**

ALI-cultured human airway epithelial cells challenged with HRV are a useful translational model for the study of HRV-induced responses in airway epithelial cells, given that gene expression profile using this model largely recapitulates some important patterns of gene responses in patients during clinical HRV infection. Furthermore, our data emphasize that both abnormal airway epithelial structure and inflammatory signaling are two important asthma signatures, which can be further exacerbated by HRV infection.

## Introduction

Rhinovirus (HRV) is not only a pathogen for the common cold, but is also the major cause of acute asthma and chronic obstructive pulmonary disease (COPD) exacerbations [1,2,3]. Virus-induced asthma exacerbations are not sufficiently controlled by currently available standard-of-care drugs [1]. An increased understanding of mechanisms linking HRV infections to asthma induction and exacerbations will provide insights for development of novel therapies for improved asthma management.

Human airway epithelial cells (HAECs) are the principal sites of HRV infection in both upper and lower airways [4]. HAECs act not only as the first line of defense against HRV, but also induce release of a wide range of mediators that drive subsequent immune and physiological responses specific to HRV [5]. Much of our current knowledge of HAEC responses to HRV infection is derived from *in vitro* HAEC culture studies. Two major approaches used to study *in vitro* responses to HRV infection include use of undifferentiated HAEC monolayers in submerged (Sub) cultures, and well-differentiated HAEC cultured in air-liquid interface (ALI) systems [6,7]. Sub cultures have been useful models for the study of HRV infection due to their ready availability, ease of culture, and facile infectability [8]. However, Sub cultures do not reproduce the structural and functional phenotype of differentiated *in vivo* airway epithelium [9], and differentiated ALI cells are considered to provide a better representation of *in vivo* airway epithelial transcription than Sub cultures (29). Differentiation of HAECs has been reported to induce resistance to HRV infection [10]. However, it is unclear whether the reported difficulty of infecting differentiated ALI cultures was due to physical reasons, such as development of physical/biochemical barriers [11], or technical reasons, such as the HRV application protocol or the relatively short time period of exposure utilized in the experiments [10].

The ability of asthmatic-derived HAEC to reproduce the asthmatic phenotype when cultured *in vitro* has been the subject of considerable recent interest. Several previously published studies indicate that asthmatic HAECs will maintain certain asthmatic features in response to HRV infection, RSV infection, air pollution, cigarette smoke or physical damage *in vitro* [12,13,14,15]. However, additional confirmation of disease phenotype maintenance is needed, particularly for passaged cells and differentiated culture models [16,17].

To gain an increased understanding of mechanisms linking HRV infection of HAECs to asthma induction and exacerbations, the goals of the current study were to evaluate ALI HAEC culture models for HRV infection, and to further identify unique gene expression patterns associated with asthmatic epithelium and HRV infection. We demonstrated efficient HRV infectability of HAEC ALI cultures, and many aspects of gene expression responses to infection that are consistent with *in vivo* human clinical findings. Previously known genes, as well as novel genes that are associated with asthma specific viral responses were identified. We conclude that ALI cultures of HAECs are useful translational models for studies of rhinovirus induced epithelial effects related to asthma exacerbation *in vitro*. Furthermore, our data suggest that abnormal airway epithelial structure and inflammatory signaling may be important contributors to viral induced asthma exacerbation.

## Materials and Methods

### HAEC culture, infection and sample preparation

Non-transplantable lungs (6 non-asthmatic and 6 asthmatic individuals) donated for medical research were obtained through the International Institute for the Advancement of Medicine (IIAM, Edison, NJ; http://www.iiam.org/) (Table 1). Signed consent to procure the tissues was obtained by IIAM. All donor identification information was removed from the sample labels by IIAM before receipt to protect the donor’s confidentiality. Donor deaths were primarily attributable to head trauma in non-asthmatic patients, whereas patients with asthma died subsequent to asthma exacerbations. Donor medical history was provided by IIAM based on physicians’ report/diagnosis and interview of family members by trained clinicians following death. Epithelial cells were isolated and cultured at passage 2 as previously described [15]. ALI cultures (0.6 cm^2^ insert devices from Millipore) were maintained in 6-well dishes containing 1 mL/well of hydrocortisone-free media for 24 hours (37°C, 5% CO_2_). The apical surface of ALI cultures was gently washed twice with 300 μL PBS to remove accumulated mucus, prior to an apical application of 50 μL vehicle (complete EMEM medium), or RV-A16 (ATCC, Manassas, VA) (5x10^5^ plaque-forming units [pfu] in 50 μL complete EMEM medium) based on an estimated *in vivo* infection dose from Chen et al.[18]. Viral titer was determined by diluting viral samples in half log units, and testing their cytopathic effects on confluent H1Hela cells (ATCC) in 24 well plates following the standard protocol [19] to obtain TCID50/mL in 5 days. The titer in pfu was calculated as 0.69xTCID50/mL, which was consistent with the titer obtained using a standard plaque assay provided by ATCC. Treated cultures were shifted to a 34°C incubator (5% CO_2_) with gentle shaking on a Bellco orbital shaker until harvested at 1.5, 8, 24 or 48 hours. Subsequent to incubation, media were harvested for secreted chemokine/cytokine assessment and immediately placed on ice, and then analyzed using a 42-plex Luminex kit (Millipore, Billerica, MA). For RNA preparation, cells were washed three times with PBS, followed by adding 175 μL RLT buffer (Qiagen) to each ALI insert. ALI cell lysates were transferred to tubes on ice, and ALI inserts were further rinsed and samples were pooled with an additional 175 μL aliquot of RLT (350 μL total/sample).

**Table 1:** Donor information.

Donor #	Asthma	Gender	Age	Race	Smoking	Height (inches)	Weight (Pounds)	Other Disease	Medications
11	No	F	38	C	Yes. 16 PY	65	106		
12	Yes	F	59	H	No	60	194	COPD, D, HTN	Advair
14	Yes	F	46	C	Yes. 30 PY	69	305	D	
15	Yes	F	45	C	Yes. 30 PY	62	165	COPD, D, HTN, G	Albuterol, Advair
18	Yes	F	64	C	No	65	189	COPD, D, HTN	Advair, Albuterol
20	No	M	13	C	No	73	220		
21	No	F	62	B	No	66	207		
22	No	M	35	C	No	68	205	HTN	Copazine, benadryl, lisinopril, darvocet
23	No	M	52	C	Yes. 20PY	69	167		
26	Yes	F	9	B	No	52	88		Albuterol, Advair
28	Yes	F	55	C	Yes. Light	65	258	D, HTN	Prednisone, Albuterol inhaler, others
30	No	M	50	H	No	72	216		

Abbreviations: F, Female; M, Male; C, Caucasian; H, Hispanic; B, Black; PY, Pack-Year, D; Diabetes, HTN; Hypertension, G; Glaucoma

### Immunohistochemistry

ALI cultured cells were fixed in 10% buffered formalin overnight, then paraffin embedded and sectioned. After antigen retrieval, slides were stained and imaged using an Olympus FluoView FV1000 confocal microscope, and analyzed using the Nikon NIS Elements software. Primary antibodies mouse anti-Muc5AC (Millipore, MAB2011), mabj2 [20], or isotype matched immunoglobins were followed by Alexa Fluor conjugated goat anti-mouse and DAPI (Life Technologies).

### RNA isolation and analysis

Total RNA was isolated from RLT cell lysates using the QIAGEN RNeasy mini kit, and quantified via Nanodrop (Thermo Scientific, Wilmington, DE), and subjected to qRT-PCR analysis. qRT-PCR primers for human genes were obtained from ABI (http://www.lifetechnologies.com). RV-A16 genome was measured by qPCR using the PrimerDesign Quantification kit for human rhinovirus 16 (genesig). To determine the copies of viral RNA in cell lysates for samples from both control and RV-A16 treatments, qPCR values were compared with the standard curve generated by using the RV-A16 positive control RNA template provided with the kit.

### Data analysis for secreted protein results

A linear mixed model was fit to data for each analyte using the MIXED procedure in SAS (SAS Institute). Donor was modeled as a random effect. Within a donor, the correlation between analyte levels measured for the same treatment at adjacent time points was modeled using a 1^st^ order autoregressive covariance structure. Treatment, disease, and time, as well as their interactions, were modeled as fixed effects. Fold induction of analytes and differences between asthmatic and non-asthmatic donors were assessed by calculating the appropriate model contrasts. The significance level used for all analyses was p< = 0.05.

### RNA-seq preparation and bioinformatics analysis

RNA sequencing was performed on an Illumina HiSeq2000 platform. Library construction and sequencing were performed by the Beijing Genomics Institute (BGI) analysis pipeline (RefSeq version HG19). RNA-seq data was deposited to GEO (GSE61141). A non-parametric Spearman’s correlation test was performed for identification of genes whose expression changes have the strongest correlation with viral titer (FDR q-value ≤ 1%). Statistical significance (nominal p-values) was corrected for multiple hypotheses testing with the Bejamini and Hochberg method [21]. A linear regression analysis was used to adjust analyses for confounding variables. To identify genes with baseline differences, a Wilcoxon rank test was performed to test the difference in gene expression between asthmatic and non-asthmatic vehicle samples. The resulting p-values were not adjusted for multiple hypothesis testing because the small sample size provided fairly limited detection power (with only 579 genes showing p-value < 0.05). Although some of these genes could be false positives due to multiple testing, many of them do appear to be functionally relevant. Gene expression pathway analysis was performed using Gene Ontology and MSigDB with hypergeometric test [22,23].

## Results

### RV-A16 infection of HAECs in ALI cultures

**Fig 1:**
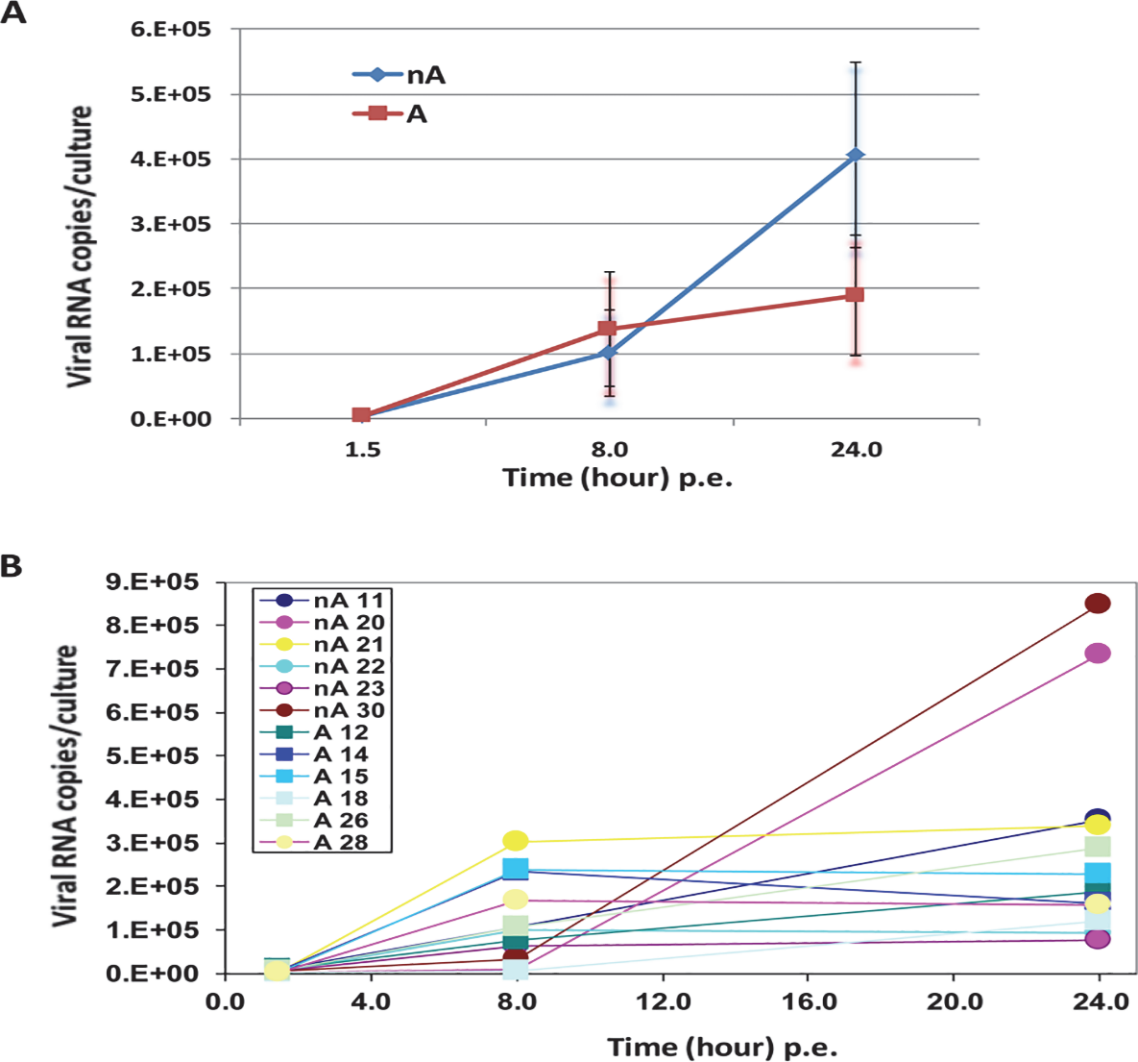
Time course of RV-A16 infection for asthmatic (n = 6) and non-asthmatic (n = 6) donor groups (A) and each donor (B) in ALI cultures. Viral genome levels in cell lysates were measured by qPCR at three time points, and were presented as viral RNA copies /culture. There was no significant difference in the mean viral genome levels between asthmatic and non-asthmatic samples. The p values between asthma and non-asthmatic samples are 0.48, 0.53 and 0.13 at 1.5, 8, and 24 hours post exposure (p.e.) respectively.

**Fig 2:**
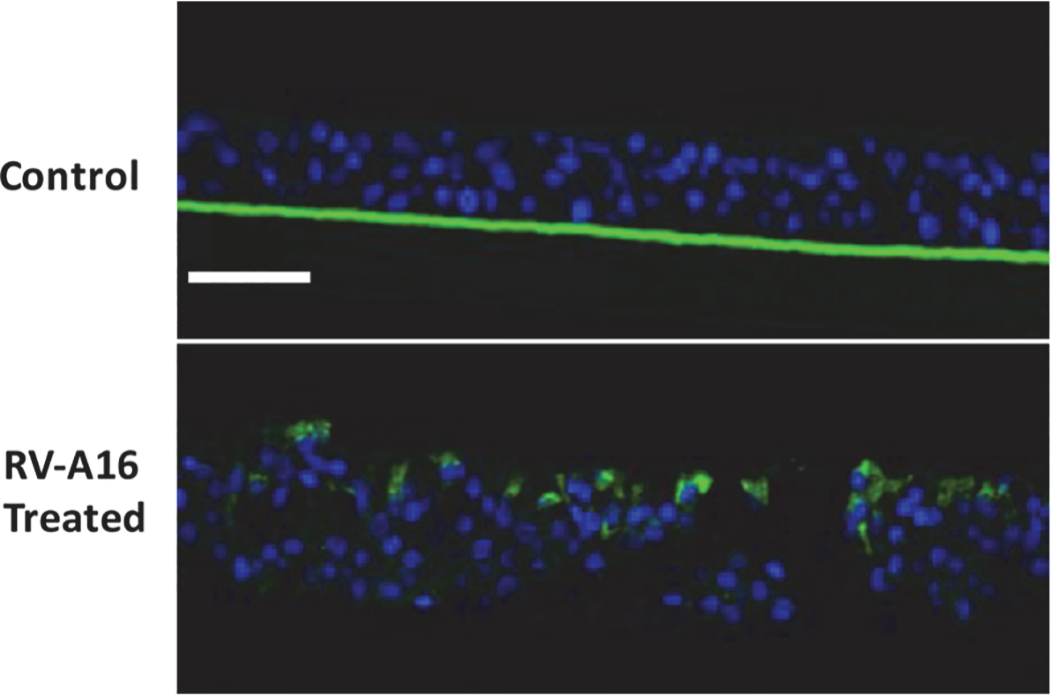
ALI cultured HAECs were infectible by RV-A16. Immunofluorescence micrographs of transverse sections of ALI cultures shows RV-A16 replicated in ALI cultured HAECs. Positive immunofluorescence staining for mabj2 (in green) was not observed in uninfected control tissues (top panel), but was observed in RV-A16 infected tissues (bottom panel); DAPI staining in blue shows cell nuclei in the tissues. Scale bars: 50 μm.

We first investigated the infectivity of RV-A16 in ALI cultured HAECs by quantifying RV-A16 viral load (RNA) in cell lysates at 1.5, 8 and 24 hours post exposure (p.e.) using qRT-PCR. Passage 2 HAECs obtained from 6 asthmatics and 6 non-asthmatics (Table 1), cultured as differentiated multilayer ALI cultures, were infected with either RV-A16 at 5x10^5^ pfu in 50 μL medium or vehicle control (medium alone) on the apical surface (see Methods). Results showed time-dependent viral load increases, which reached maximum at 24 hours p.e. (Fig. 1A), indicating that ALI culture systems are effectively infected by RV-A16. Cell lysates at 48 hours p.e. were not included in the analysis, as viral load was significantly decreased in cell lysate, due to rhinovirus shedding from cells into culture medium at this time point. We noted that, unlike cells from other donors, ALI cultured HAECs from non-asthma donor 23 and asthma donors 14 and15 had a decreased viral load from 8 hour to 24 hour p.e. (Fig. 1B). However, no statistically significant differences in viral load were observed between asthmatic and non-asthmatic derived cultures (Fig. 1A). Anti-dsRNA antibody J2 (mabJ2) [20] was utilized to detect replicating HRV genomes by immunofluorescence staining. Positive staining was observed at the apical side of ALI cultured HAECs, confirming that ALI cultures were infectable and enabled HRV replication (Fig. 2).

### Genome-wide gene expression profiles of ALI HAEC cultures following RV-A16 infection

We next utilized genome-wide RNA-seq analysis to investigate whether non-asthmatic and asthmatic ALI HAEC cultures exhibited differential gene expression at baseline, and/or after HRV infection. ALI cultures infected by RV-A16 at 24 hours p.e were chosen for the analysis because our analysis of expression changes of selected genes at different time points after viral infection indicated that the most robust responses were induced by RV-A16 at 24 hours p.e., which also correlated with the highest viral content in cells (not shown).

**Fig 3:**
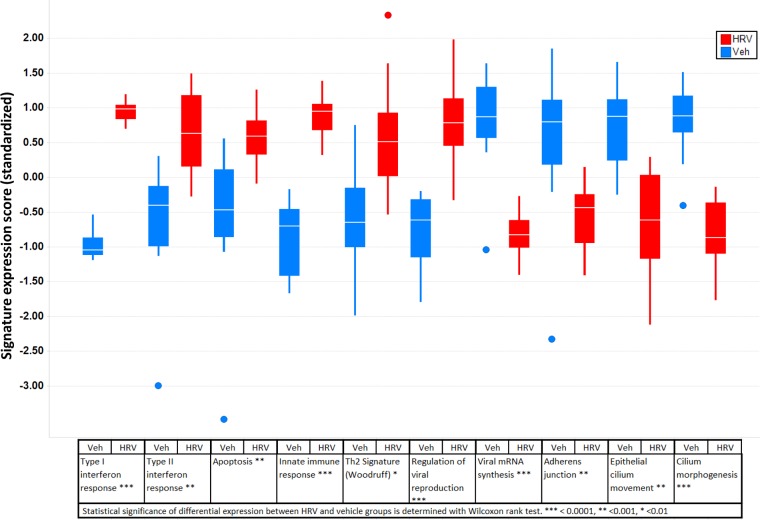
Functional pathways that showed statistically significant differential expression between RV-A16 and Vehicle groups (p<0.05). Outliner is shown individually with a solid circle.

To identify genes whose expression was affected by viral infection, we performed a non-parametric Spearman’s correlation test on RNA-seq gene expression data from the samples treated with vehicle or RV-A16 across all 12 donors. We identified 1,485 genes that had strongest correlation with viral infection (FDR q-value ≤ 1%), among which 457 were up-regulated (see S1 Dataset). Gene expression pathway analysis of these genes identified 10 functional pathways that showed statistically significant differential expression between RV-A16 and vehicle groups (Fig. 3). Genes from 4 pathways, including those involved in mRNA synthesis, adherens junction, epithelial cilium movement and cilium morphogenesis, had significantly down-regulated expression in RV-A16 infected cells compared to vehicle treated cells (Fig. 3). This finding is consistent with host cell transcription shut off, disruption of HAEC polarity, and ciliated cell dysplasia due to RV-A16 infection. An additional 6 pathways that were significantly increased after viral infection were involved in type I and type II interferon responses, apoptosis, innate immune responses, a Th2 signature from Woodruff [24,25] and pathways for regulation of viral production (Fig. 3 and S2 Dataset).

We further compared the genome-wide RNA-seq expression changes observed in our RV-A16-infected ALI cultures to *in vivo* gene signature data obtained from nasal scrapings at 48 hours after RV-A16 challenge as previously reported by Proud and coworkers [26]. There are 452 up-regulated genes in our study that map to Refseq genes, and 381 upregulated genes in the Proud study. The overlap is 61 genes (p = 2.2e-28) (S3 Dataset). GeneGO pathways analysis revealed that pathways enriched in these genes are immune response (p = 2.575E-20), defense response to virus (p = 7.387E-20) and interferon signaling (p = 2.955E-18). In addition, there are 1041 down-regulated genes in our dataset (mapped to Refseq), and 256 in the Proud study. The overlap is 35 (p = 2.3E-5) (S3 Dataset), which enriched Th2 signature in lung epithelial brushings from Woodruff studies (p = 0.026) [24,25].

### Asthma-specific baseline gene expression profiles in ALI cultured HAECs

To investigate whether asthmatic HAECs still maintained a distinct gene expression profile from non-asthmatic cells in ALI cultures, a non-parametric Wilcoxon-test was used to compare the rank-order of gene expression between vehicle treated samples from two donor groups (asthma vs. non-asthma). Although the magnitude of gene expression differences between asthma and non-asthma groups was overall quite low, a total of 579 genes were identified as differentially expressed with statistical significance (p<0.05). This list includes 346 with increased expression, and 233 with reduced expression in vehicle treated asthmatic compared to non-asthmatic cultures (S4 Dataset). Function pathway analysis revealed a strong Th-2 signature and cilium function signature in asthmatic samples. Twenty-three genes with reduced expression in asthmatic samples coincided with the down-regulated arm of a Th-2 signature generated in lung epithelial brushings as reported previously by Woodruff *et al* [24,25] (S5 Dataset). This finding suggests that asthmatic donors in this study primarily may be Th-2 type. Additionally, 12 down-regulated genes were related to cilium function, demonstrating that gene signature of ciliary dysfunction, a feature of moderate to severe asthma pathophysiology [27], was preserved in ALI cultures even after 2 passages *in vitro*.

### Asthmatic-specific viral response gene expression profiles in ALI cultured cells

**Fig 4:**
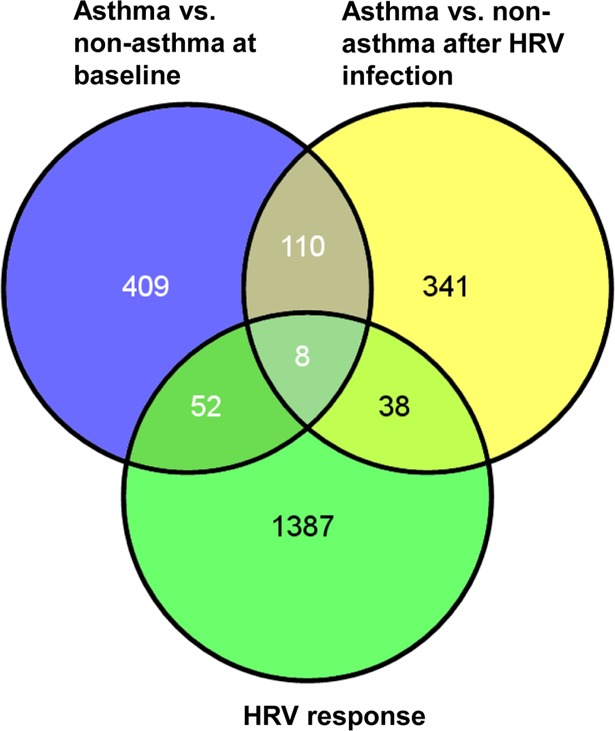
Distributions of differentially expressed genes in asthmatic samples after treatment of vehicle or RV-A16. 579 genes in the “Asthma vs. non-asthma at baseline” group had baseline difference between asthmatic and non-asthmatic cells (p<0.05); 497 genes in the “Asthma vs. non-asthma after HRV infection” group had asthma-related viral response (p<0.05); and 1485 genes in the “HRV response group had strong expression changes associated with viral infection across both asthma and non-asthma donors. The numbers of genes only in one group or common for either two or three groups are shown by either exclusive or overlapping areas in Venn diagrams. The diagrams were generated with an online tool available at http://bioinfogp.cnb.csic.es/tools/venny/.

We next sought to identify genes that responded to HRV differently between asthmatic and non-asthmatic groups. By fitting a linear mixed-effects model, we identified a total of 497 genes with nominally significant difference (p< 0.05) in viral infection response between asthmatic and non-asthmatic groups (S6 Dataset). Of these, 46 genes had expression changes strongly associated with RV-A16 infection (Fig. 4). Notably, 8/46 genes also showed differential expression at baseline in the vehicle treated cells, indicating these genes were intrinsically different in expression between asthmatic and non-asthmatic donors prior to viral infection (Table 2). For example, chemokine (C-C motif) ligand 5 (CCL5), also known as RANTES, previously reported to be genetically associated with asthma [28,29], is differentially expressed between asthmatic and non-asthmatic cultures at both baseline (p = 0.026) and post viral infection (p = 0.047). Its expression increased 1.86 fold after viral infection and the increase highly correlated with viral load (correlation co-efficient = 0.982). 38/46 genes showed strong differential expression associated with asthma-related viral infection, but without baseline difference in expression prior to viral infection (Table 3). Notably, the expression of CXCL10 (IP10), a chemoattractant for type I lymphocyte and NK cells, and previously proposed as a biomarker of human HRV infection and exacerbation of COPD [30], significantly increased 3.4 fold higher in asthmatic cells after viral infection, compared to non-asthmatic cells (p = 0.005). Our analysis identified 52 genes that showed differential expression between asthma and non-asthma donor cells at the baseline, but not after viral infection, although they were strongly responsive to viral infection (Table 4). Interestingly, the changes in expression of lectin galactoside-binding soluble 9 (LGALS9) [31], chemokine (C-X3-C motif) ligand 1 (CX3CL1) [32] and complement component 5a receptor 1 (C5AR1) [33] have been previously reported to be associated with asthma. Cadherin related family member 3 (CDHR3) was recently reported to be genetically associated with early childhood severe and recurrent asthma exacerbation, and highly expressed in airway epithelium [34]. CDHR3 encodes a hemophilic cell adhesion molecule, which may be involved in maintaining cell integrity by forming cell-cell junctions. In this study, we found its expression was significantly lower in asthmatic epithelial cells compared to non-asthmatic cells at the baseline (p = 0.02) (S4 Dataset), consistent with the previous report that showed its expression was down-regulated in allergic asthma subjects [24] (S5 Dataset). Importantly its expression was further decreased upon viral infection (HRV vs. Veh p<0.0001) and its reduction was highly correlated with viral infection (Pearson correlation p value = 1.6e-04 and q value = 2.944e-03) (S1 Dataset). Our data provide evidence for further disrupted function of CDHR3 in HRV-induced asthma exacerbation.

**Table 2:** Differentially expressed genes between asthma and non-asthma groups both at baseline and after HRV 16 infection.

Gene Symbol	Gene name	Biological function	Base line difference p-value	Differential viral response (As vs. non-As)		Viral infection correlation p-value
				Fold Change	p-value	
CCRL1	chemokine (C-C motif) receptor-like 1	decoy chemokine receptor	0.002	1.686	0.049	4.1E-06
LRIG1	Leucine-rich repeats and immunoglobulin-like domains protein 1	protein binding	0.004	0.528	0.016	2.8E-07
ERBB4	v-erb-a erythroblastic leukemia viral oncogene homolog 4 (avian)	a receptor tyrosine kinase	0.009	1.358	0.037	2.6E-07
RNF44	ring finger protein 44	protein binding	0.009	0.701	0.044	2.7E-06
STARD4	StAR-related lipid transfer (START) domain containing	lipid transport	0.015	1.857	0.013	6.6E-10
CCL5	chemokine (C-C motif) ligand 5	chemokine	0.026	1.857	0.047	2.6E-14
LOC388692	hypothetical LOC388692	NA	0.026	1.432	0.043	7.4E-07
KLHDC3	kelch domain containing 3	protein binding	0.041	0.581	0.028	2.1E-06

**Table 3:** Differentially expressed genes between asthma and non-asthma groups after HRV 16 infection.

Gene Symbol	Gene name	Biological function	Differential viral response (As vs. non-As)		Viral infection correlation p-value
			Fold change	p-value	
CXCL10	chemokine (C-X-C motif) ligand 10	chemokine	3.353	0.005	9.5E-15
ZBP1	Z-DNA binding protein 1	DNA binding	1.522	0.050	2.9E-15
C19orf66	chromosome 19 open reading frame 66	NA	1.402	0.046	1.2E-14
GBP5	guanylate binding protein 5	Guanylate binding protein	1.634	0.035	9.5E-13
NUB1	negative regulator of ubiquitin-like proteins 1	protein binding	1.548	0.030	1.3E-12
C1orf38	chromosome 1 open reading frame 38	NA	1.891	0.012	7.1E-12
TICAM1	toll-like receptor adaptor molecule 1	protein binding, anti-viral defense, apoptosis	1.477	0.043	6.5E-12
APOBEC3F	apolipoprotein B mRNA editing enzyme, catalytic polypeptide-like 3F	protein binding, RNA binding, anti-viral defense	1.558	0.035	4.1E-10
BCL7A	B-cell CLL/lymphoma 7A	negative regulation of transcription	0.513	0.017	6.9E-08
FAM104B	family with sequence similarity 104, member B	NA	0.592	0.049	3.0E-08
CCDC109B	coiled-coil domain containing 109B	ion channel activity	1.844	0.043	2.1E-07
PARP8	ubiquitin specific peptidase 15	NA	1.556	0.021	7.0E-08
GCLC	glutamate-cysteine ligase, catalytic subunit	glutamate cystein ligase activity	0.654	0.022	1.4E-06
FAM60A	family with sequence similarity 60, member A	protein binding	0.624	0.007	8.4E-08
KCNQ4	potassium voltage-gated channel, KQT-like subfamily, member 4	ion channel activity	2.106	0.026	2.1E-06
VGLL4	vestigial like 4 (Drosophila)	transcription	0.701	0.032	1.3E-07
GOLPH3L	golgi phosphoprotein 3-like	protein binding	0.681	0.037	2.2E-06
LIMS1	LIM and senescent cell antigen-like domains 1	protein binding	0.543	0.004	1.1E-06
USP15	ubiquitin specific peptidase 15	enzyme activity	1.453	0.035	1.2E-06
PEX6	peroxisomal biogenesis factor 6	protein binding	0.555	0.045	4.9E-06
FHOD1	formin homology 2 domain containing 1	protein binding, actin binding	0.592	0.044	6.0E-06
FXR1	fragile X mental retardation, autosomal homolog 1	RNA binding	0.709	0.020	1.2E-06
IARS2	isoleucyl-tRNA synthetase 2, mitochondrial	ATP binding, aminoacyl -tRNA editing activity	0.708	0.016	2.7E-06
PTPLAD2	protein tyrosine phosphatase-like A domain containing 2	lysase activity	0.426	0.010	6.1E-05
SLC23A2	solute carrier family 23 (nucleobase transporters), member 2	nucleobase transporter	0.670	0.048	1.0E-05
PELI1	pellino homolog 1 (Drosophila)	protein binding, ubiquitin-protein ligase activity	1.754	0.020	1.1E-05
TMCC2	transmembrane and coiled-coil domain family 2	protein binding	0.490	0.013	6.9E-06
LRRC3	leucine rich repeat containing 3	protein binding	1.671	0.030	2.7E-05
DHX32	DEAH (Asp-Glu-Ala-His) box polypeptide 32	helicase activity, ATP binding	0.601	0.049	5.7E-05
IVD	isovaleryl-CoA dehydrogenase	metabolic pathway	0.643	0.043	2.4E-05
ZFHX2	zinc finger homeobox 2	transcription	0.606	0.022	4.4E-05
PDK2	pyruvate dehydrogenase kinase, isozyme 2	metabolic pathway	0.482	0.023	4.4E-05
RBBP4	retinoblastoma binding protein 4	protein binding, histone binding	0.758	0.044	2.3E-06
VAT1	vesicle amine transport protein 1 homolog (T. californica)	transporter	0.568	0.029	2.9E-05
CACNA1D	calcium channel, voltage-dependent, L type, alpha 1D subunit	ion channel activity	0.705	0.046	3.9E-05
TTLL1	tubulin tyrosine ligase-like family, member 1	cilium function	0.492	0.012	8.2E-05
ANKMY1	ankyrin repeat and MYND domain containing 1	protein binding, metal ion binding	0.491	0.047	3.7E-04

**Table 4:** Differentially expressed genes between asthma and non-asthma groups at baseline and also significantly responsive to RV-A16 infection.

Gene Symbol	Gene name	Biological function	Baseline difference P-value	Viral infection correlation p-value
BBS1	Bardet-Biedl syndrome 1	Acetylation, cilia assembly	0.0022	2.7E-08
LGALS9	lectin, galactoside-binding, soluble, 9	Cell adhesion	0.0043	7.1E-11
BAZ1A	bromodomain adjacent to zinc finger domain, 1A	Transciption	0.0087	4.6E-08
CX3CL1	chemokine (C-X3-C motif) ligand 1	Chemokine	0.0087	3.5E-09
KIAA0232	KIAA0232	Unknown	0.0087	5.5E-08
SAP30	Sin3A-associated protein, 30kDa	Transciption	0.0087	5.6E-08
APOBEC4	apolipoprotein B mRNA editing enzyme, catalytic polypeptide-like 4 (putative)	mRNA editing	0.0152	3.1E-06
BTN3A3	butyrophilin, subfamily 3, member A3	T cell mediated immunity	0.0152	1.5E-07
CCDC30	coiled-coil domain containing 30	Unknown	0.0152	7.4E-05
FAM81A	family with sequence similarity 81, member A	Unknown	0.0152	2.7E-06
IFI30	interferon, gamma-inducible protein 30	Superoxide anion generation	0.0152	1.7E-09
KIAA0319	KIAA0319	Cell adhesion	0.0152	1.2E-05
KLC4	kinesin light chain 4	Microtubule motor activity	0.0152	1.0E-06
MARCKSL1	MARCKS-like 1	Actin binding	0.0152	4.6E-08
NEK1	NIMA (never in mitosis gene a)-related kinase 1	Cilium assembly	0.0152	3.9E-06
PRSS12	protease, serine, 12 (neurotrypsin, motopsin)	Zymogen activation, exocytosis	0.0152	2.2E-07
RAPGEF5	Rap guanine nucleotide exchange factor (GEF) 5	Small GTPase mediated signal transduction	0.0152	1.8E-08
WDR52	WD repeat domain 52	Unknown	0.0152	1.8E-05
WRB	tryptophan rich basic protein	Unknown	0.0152	3.1E-08
C1orf88	chromosome 1 open reading frame 88	Cell projection organization	0.0260	2.2E-08
CDHR3	cadherin-related family member 3	Homophilic cell adhesion	0.0260	1.6E-04
CHKA	choline kinase alpha	Lipid metabolic process	0.0260	2.4E-06
CLOCK	clock homolog (mouse)	Transciption	0.0260	5.9E-07
DAB2IP	DAB2 interacting protein	Negative regulation of cell migration	0.0260	6.2E-08
DNAH12	dynein, axonemal, heavy chain 12	Microtubule motor activity	0.0260	2.4E-05
ENDOD1	endonuclease domain containing 1	Unknown	0.0260	1.3E-11
EPB41L4B	erythrocyte membrane protein band 4.1 like 4B	Actomyosin structure organization	0.0260	3.8E-06
GNL3L	guanine nucleotide binding protein-like 3 (nucleolar)-like	GTP catabolic process; ribosome biogenesis	0.0260	2.0E-06
HIST2H2BE	histone cluster 2, H2be	Nucleosome assembly	0.0260	2.5E-05
KIAA1377	KIAA1377	Unknown	0.0260	9.7E-06
LRRC6	leucine rich repeat containing 6	cilium movement	0.0260	3.2E-04
NRG2	neuregulin 2	Signal transduction	0.0260	5.5E-05
PCM1	pericentriolar material 1	Cilium assembly	0.0260	5.4E-07
SPATA6	spermatogenesis associated 6	Cell differentiation	0.0260	9.9E-07
SPICE1	spindle and centriole associated protein 1	Cell division	0.0260	6.9E-08
TCP11L2	t-complex 11 (mouse)-like 2	Cell morphogenesis	0.0260	1.3E-06
TMEM67	transmembrane protein 67	Cilium assembly	0.0260	6.1E-07
C5AR1	complement component 5a receptor 1	Epithelial cell proliferation	0.0260	6.4E-08
CERKL	ceramide kinase-like	Negative regulation of cell Apoptosis	0.0411	3.9E-06
CNNM3	cyclin M3	Ion transport	0.0411	5.2E-07
DNAH6	dynein, axonemal, heavy chain 6	Microtubule-based movement	0.0411	4.0E-05
DNAL1	dynein, axonemal, light chain 1	Cilium function	0.0411	4.9E-07
ENKUR	enkurin, TRPC channel interacting protein	Cilium function	0.0411	8.0E-06
GPR85	G protein-coupled receptor 85	Signal transduction	0.0411	1.8E-09
IFITM1	interferon induced transmembrane protein 1 (9–27)	Negative regulation of viral genome replication	0.0411	1.9E-10
KIF2A	kinesin heavy chain member 2A	Microtubule-based movement	0.0411	5.8E-09
LOC157381	hypothetical LOC157381	Unknown	0.0411	2.2E-06
LOC646851	similar to OTTHUMP00000028720	T cell proliferation	0.0411	2.0E-05
RABGAP1L	RAB GTPase activating protein 1-like	Regulation of protein organization	0.0411	2.0E-05
RABL5	RAB, member RAS oncogene family-like 5	Small GTPase mediated signal transduction	0.0411	4.0E-07
SYBU	syntabulin (syntaxin-interacting)	Unknown	0.0411	2.4E-08
PSMB9	proteasome (prosome, macropain) subunit, beta type, 9 (large multifunctional peptidase 2)	Cell cycle	0.0450	5.7E-07

### Cytokine/chemokine expression changes induced by RV-A16 infection

**Fig 5:**
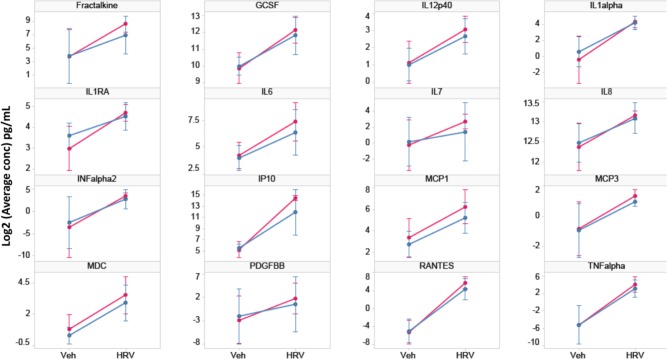
Expression of secreted cytokines/chemokines in culture medium. 16/42 cytokines tested had robust expression levels and were significantly up-regulated at 24 hr after RV-A16 infection across 12 donors (p value<0.05). Differences were not significant between asthma and non-asthma groups (p>0.05). Red lines represent asthma samples, blue lines represent non-asthma samples. Each line represents the mean and standard deviation of log2 concentration of each cytokine/chemokine.

To compare protein expression of common cytokines and chemokines induced by HRV between asthmatic and non-asthmatic cells, we measured their expression level in culture medium using a Luminex 42-plex assay. The expression level of 3 analytes (EGF, FGF2, and IL4) was below detection, and 7 analytes (GMCSF, GRO, IL15, IL1beta, IL3, PDGRA and VEGF) were not significantly induced by RV-A16. Thus, these proteins were not included in further analysis. Thirty-two cytokines/chemokines showed significant expression changes upon viral infection (S7 Dataset), among which 16 analytes had robust expression levels (Fig. 5). However, none of them showed any difference between asthmatic and non-asthmatic cells (S7 Dataset). Generally, there is a good agreement between proteins and their corresponding genes in terms of their expression change directions (i.e. proteins up-regulated by HRV tend to be up-regulated at the mRNA level).

### HRV infection induced MUC5AC expression in ALI cultures

**Fig 6:**
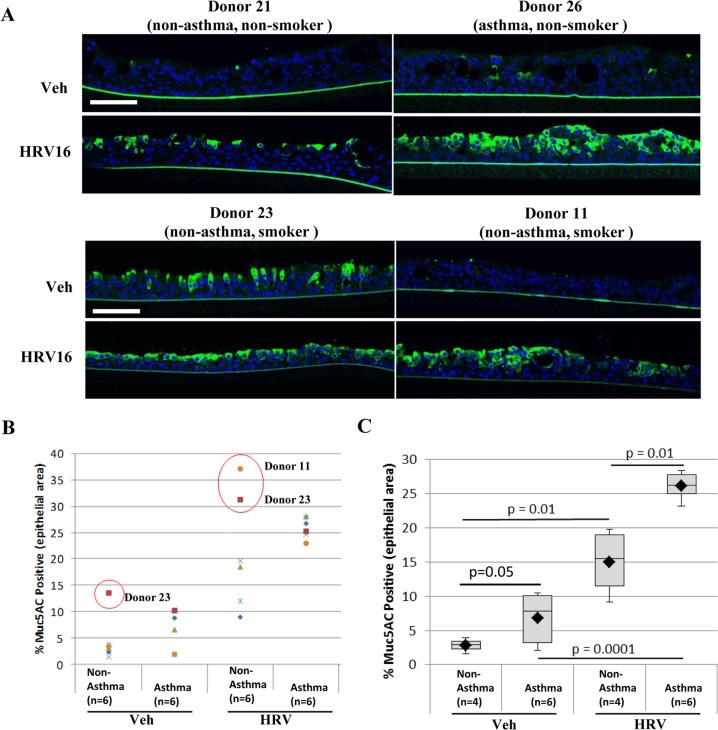
MUC5AC expression is upregulated in response to HRV infection. A) Immunofluorescence of MUC5AC in ALI cultures from donors 21 (non-asthma, non-smoker), 23 (non-asthma, smoker), 26 (asthma, non-smoker) and 11(asthma, smoker) show an increase in staining with HRV treatment, with only donor 23 having high baseline expression. Green: MUC5AC, blue: DAPI staining for nuclei, representative images, scale bar: 50 μm. N = 6 ALI cultures per group (asthma and non-asthma). B and C) Quantification of immunofluorescence staining presented as either dot plot (B) or box plot (C). Positively stained area was measured and presented as % of total epithelial area. Red dots indicate outliers (> 1.5x inter-quartile distance). B) Inclusion of all data from 6 non-asthmatic and 6 asthmatic donors in analysis. C) Removal of non-diseased smokers (donors 11 and 23) from the data set results in significant differences between HRV and vehicle treated tissues in both non-asthmatic and asthmatic donors as determined by ANOVA and Tukey HSD test.

As mucus hypersecretion relates to HRV-induced asthma exacerbations, MUC5AC protein expression was examined in ALI cultures after RV-A16 infection compared to vehicle treatment by immunofluorescence (Fig. 6). We observed that basal levels of its expression in vehicle treated tissues were generally higher in non-asthma smokers and asthma donors compared to non-asthma, non-smoker donors, and that its expression level was increased after viral infection (Fig. 6A and B). However there was no statistical significance in terms of these differences or asthma-related expression changes (Fig. 6B). Smoking has previously been linked to increased mucus production and MUC5AC gene expression [35]. We found that non-asthma smoker donor 23 had three fold higher basal expression level of MUC5AC, whereas non-asthma smoker donor 11 had an expression level of MUC5AC similar to other non-asthma non-smoker donors (Fig. 6A). Removal of non-asthma smokers (donors 11 and 23) from the data set resulted in significant responses of MUC5AC expression after RV-A16 infection in both non-asthma donors (p = 0.01) and asthma donors (p = 0.0001). Importantly, MUC5AC protein expression level showed asthma-related differences both at vehicle treated baseline (p = 0.05) and after viral infection (p = 0.01) (Fig. 6C). The MUC5AC gene initially was not included in the gene annotation used to quantitate transcription in RNA-seq analysis. There are, however, reads mapped to the MUC5AC locus. Its FPKM was recalculated using a different genome annotation to quantitate its expression levels. Interestingly, consistent with MUC5AC protein expression results, base line MUC5AC mRNA expression was highest in the non-asthma smoker donor 23 (S1 Fig.). When both non-asthma smoker donors 23 and 11 were removed from analysis, MUC5AC gene expression level showed both significant response to RV-A16 infection (p = 0.016 and p = 0.004 for non-asthma and asthma groups respectively), and asthma-related differential viral response between the asthma and non-asthma groups (p = 0.047) (S2 Fig.).

## Discussion

Some previous studies reported that differentiated HAECs were more resistant to HRV infection compared to undifferentiated cells, because only fewer than 5% of the differentiated cells were infected [10][18]. However, using our current infection protocol, we found that differentiated ALI cultured HAECs can be effectively infected by RV-A16, based on quantitative analysis of viral RNA in cell lysates (Fig. 1) and IHC analysis for viral replication dsRNA intermediates that showed most apically located cells were infected (Fig. 2). This discrepancy might result from the different duration of viral exposure on HAECs. In this study, we tried to mimic the *in vivo* situation where once HRV gets into the respiratory tract, the epithelium is exposed to viral infection for an extended period of time until possible complete or partial clearance. Mucus was also rinsed from the surface of ALI prior to HRV application in the current study to allow better attachment of HRV to epithelial cells. Thus, RV-A16 was left in the culture until cells were harvested for analysis, whereas previously reported studies only allowed 1 to 6 hr viral exposure, then virus was removed from culture [10,18]. We speculate that ALI cultured HAE cells may require a longer time for effective HRV infection. This may be due to low numbers of ICAM-1 receptors on the membranes of apical surface cells in ALI cultures [36]. Some other studies have also successfully shown HRV infection in ALI cultured airway epithelial cells [37,38,39,40], and demonstrated optimal replication of some strains from RV-A and RV-C classes can be achieved only when the virus is added apically [40], emphasizing the importance of polarized ALI cultures for viral infection study.

We observed robust responses in ALI cultured HAECs elicited by RV-A16 infection. The RNA-seq analysis identified 1485 genes that are significantly responsive to viral infection in HAEC ALI cultures at 24 hours post exposure. This set of genes included all the anti-viral pathway components identified by Chen et al. in non-asthmatic ALI cultured airway epithelial cells [18]. Importantly, these 1485 genes also showed a significant overlap with those from the *in vivo* dataset previously reported by Proud and coworkers [26] (S3 Dataset), demonstrating that some important patterns of gene expression changes observed in nasal scrapes during clinical RV-A16 infection in subjects can be reproduced in the ALI cultured second passage HAECs, in spite of differences between cell types in these two studies. Taken together, we conclude that the ALI cultured HAECs infected with HRV represent a translational *in vitro* model for capturing *in vivo* rhinoviral infection responses. Our ALI culture model is composed of epithelial cells, but lacks immune system cells such as dendritic cells, macrophages, T cells, eosinophils, which are known to be important players in viral sensing, amplification of antiviral response and allergic inflammation in airways [3]. Studies using blood cells [41] or a coculture system including both HAECs and other cell types such as dendritic cells, and/or lymphocytes should allow us to gather a more complete picture of HRV infection responses that occur *in vivo*.

The major limitation of this study is a relatively small number of samples (6 asthmatic, 6 non-asthmatic cells), which have heterogeneous characteristics such as smoking history, and other diseases (Table 1). Nevertheless, we observed significant, strong asthma specific Th-2 and cilium function signatures in control ALI cultures (S4 Dataset). These signatures are consistent with features of moderate to severe asthma pathophysiology previously described *in vivo* [24,25,27]. Collectively, these results demonstrate that common asthmatic features may still be identified by gene expression signatures despite small sample size and a heterogeneous background of donor samples. In addition, we noted that cells derived from non-asthmatic donor 23 behaved differently from other non-asthmatic samples in some respects. For example, donor 23 cells were less infected by RV-A16 at 24 hrs (Fig. 1), despite having similar levels of ICAM1 gene expression compared to other non-asthmatic donor cells before and after RV-A16 infection (S1 Dataset). It is unclear if donor 23 cells behaved differently from other non-asthmatics simply due to smoking history (20PY), since cells from donor 11, also a smoker (16PY), did not display the same resistance to viral infection. Smoking has previously been linked to increased mucus production and MUC5AC gene expression [35]. Consistent with this, the ALI culture derived from donor 23 had a higher amount of MUC5AC expression before viral infection (Fig. 6), but donor 11 had a similar expression level to other non-asthma donors. Recently, the study from Jakiela et al. demonstrated that IL13-induced mucous metaplasia rendered ALI cultured airway epithelial cells less susceptible to HRV infection [38]. It is likely that relatively highly increased mucus production may render donor 23 cells less infectable by HRV. Further study on the genetic background of this donor is warranted to understand this phenotype. Nevertheless, due to the relatively small sample size of this study, we included donor 23 in the non-asthmatic group for all analyses.

Conflicting reports exist in the literature regarding defective HRV-induced type I and type III interferon production or increased viral infection in primary HAECs derived from asthma subjects [12,42,43]. The reasons for this are unclear, but is likely influenced by differences [8] in experimental techniques (ALI versus submerged cultures) and clinical characteristics of asthmatic subjects from whom the primary HAECs were derived (severe asthmatic subjects in our study versus mild atopic asthmatic donors in others) [8,44]. We did not observe any significantly differential expression of type III interferon genes (IL28A, IL28B and IL29), or type I interferon gene IFNB1 related to asthma, although they showed robust responses to RV-A16. In addition, we did not observe viral load differences between asthmatic and non-asthmatic cells, which was in good agreement with the recent clinical studies from Kennedy et al., which showed that individuals with and without asthma had similar viral loads after RV-A16 infection [45]. Similarly, protein expression of IL6 and IL8 was significantly induced upon viral infection, but no significant differential expression was observed between asthma and non-asthma groups.

Among the 1485 genes associated with HRV infection, three groups were categorized based on differential expression changes either at baseline or after RV-A16 infection between asthmatic and non-asthmatic cells. The first group included 8 genes with differential expression both at baseline and after RV-A16 infection. The second group included 38 genes with differential expression only after viral infection, and the third group had 52 genes with differential expression only at baseline (Fig. 4). Interestingly, genes previously associated with asthma, such as CCL5 (RANTES), CXCL10 (IP10), LGALS9, CX3CL1, C5AR1, and CDHR3 fall into these three different groups. Other genes in these three groups included several involved in chemokine signaling such as CCRL1. Notably, many are involved in epithelial cell remodeling and cilia assembly and function, most of which belonged to the third group and also were found as part of Th2 signature in lung epithelial brushings from Woodruff studies, and cilium signature from Swiss Protein keywords (LRIG1, KLHDC3, ERBB4, FHOD1, TTLL1, BBS1, KIAA0319, NEK1, C1orf88, DAB2IP, DNAH12, EPB41L4B, LRRC6, PCM1, TCP11L2, TMEM67, DNAH6, DNAL1, ENKUR, KIF2A). These findings provide evidence that ALI cultured HAECs derived from asthmatic donors have fundamental structural differences at baseline in the absence of infection, and these differences can be further exacerbated by RV-A16 infection at least by gene expression standards. It is important to note that, although differences in gene signatures of immune and defense responses between asthma and non-asthma cells were commonly identified in both ALI and submerged cultures, these structural gene expression differences were not revealed when undifferentiated submerged cultures were used for study [8].

Taken together, we have demonstrated that ALI cultured HAECs can be effectively infected with RV-A16, and produce robust responses. Given that 1) ALI has morphological and structural features associated with differentiated cells; 2) ALI culture can be infected with HRV using the protocol reported in the current study; and 3) as demonstrated here, the *in vitro* viral response gene expression changes aligns with patient data as recently reported [24,26,44], we propose that ALI cultured HAECs be a sensitive translational model to study HRV-induced changes in HAECs. Furthermore, using RNA-seq, we identified unique asthma-specific expression patterns both at baseline and after RV-A16 infection in ALI cultured HAECs. Besides inflammatory signaling, these differential expression patterns were largely structurally related, and involved in epithelial cell remodeling, cell-cell junctions and cilium assembly and function. These findings provide additional evidence that abnormal airway structure, physiology and inflammatory conditions are important contributors to asthma, which may be further exacerbated by HRV infection.

## Supporting Information

S1 Fig A dot plot of MUC5AC RNA-seq expression in 12 donors after treatment of vehicle (Veh) or RV-A16 (HRV) for 24 hours. MUC5AC mRNA expression level based on RNA-seq reads was quantitated using a different annotation genome. Non-asthma donor 23 had the highest expression of MUC5AC at baseline and after HRV infection.
(PDF)

S2 Fig A box plot of MUC5AC RNA-seq expression in 10 donors after treatment of vehicle (Veh) or RV-A16 (HRV) for 24 hours. Removal of non-diseased smokers (donors 11 and 23) from the data set results in significant differences between HRV and vehicle treated tissues in both non-asthmatic and asthmatic donors, and significant differential responses to HRV between asthma and non-asthma groups as determined by ANOVA and Tukey HSD test.
(PDF)

S1 Dataset A list of 1485 genes whose expression changes are strongly correlated with viral infection. (XLSX)

S2 Dataset Lists of genes in 10 functional groups that show statistically significant differential changes in gene expression after viral infection. (XLSX)

S3 Dataset Lists of RV-A16 responsive genes that are overlapped between *in vitro* HAECs from the current study and *in vivo* nasal scrapings reported in the Proud study. (XLSX)

S4 Dataset A total of 579 genes with significant baseline differential expression in ALI cultured HAECs from asthmatic compared to non-asthmatic donors (p<0.05). (XLSX)

S5 Dataset Genes related to Th-2 signature or cilium function that have significant baseline differential expression between asthmatic and non-asthmatic ALI samples. (XLSX)

S6 Dataset A list of 497 genes that showed marginally significant difference in viral induced gene expression changes between asthmatic between non-asthmatic ALI samples (nominal p <0.05). (XLSX)

S7 Dataset A list of cytokines/chemokines in the Luminex 42-plex assay panel whose expression levels had significant changes upon viral infection. (XLSX)

## References

[1] Hershenson MB. Rhinovirus-Induced Exacerbations of Asthma and COPD. Scientifica (Cairo) 2013: 405876 10.1155/2013/405876 24278777 PMC3820304

[2] Gavala ML, Bertics PJ, Gern JE. Rhinoviruses, allergic inflammation, and asthma. Immunol Rev. 2011; 242: 69–90. 10.1111/j.1600-065X.2011.01031.x 21682739 PMC3119863

[3] Gern JE. The ABCs of rhinoviruses, wheezing, and asthma. J Virol. 2010; 84: 7418–7426. 10.1128/JVI.02290-09 20375160 PMC2897627

[4] Holgate ST. The sentinel role of the airway epithelium in asthma pathogenesis. Immunol Rev. 2011; 242: 205–219. 10.1111/j.1600-065X.2011.01030.x 21682747

[5] Holgate ST. Mechanisms of Asthma and Implications for Its Prevention and Treatment: A Personal Journey. Allergy Asthma Immunol Res. 2013; 5: 343–347. 10.4168/aair.2013.5.6.343 24179679 PMC3810539

[6] Whitcutt MJ, Adler KB, Wu R. A biphasic chamber system for maintaining polarity of differentiation of cultured respiratory tract epithelial cells. In Vitro Cell Dev Biol. 1988; 24: 420–428. 3372447 10.1007/BF02628493

[7] Yamaya M, Finkbeiner WE, Chun SY, Widdicombe JH. Differentiated structure and function of cultures from human tracheal epithelium. Am J Physiol. 1992; 262: L713–724. 1616056 10.1152/ajplung.1992.262.6.L713

[8] Bochkov YA, Hanson KM, Keles S, Brockman-Schneider RA, Jarjour NN, Gern JE. Rhinovirus-induced modulation of gene expression in bronchial epithelial cells from subjects with asthma. Mucosal Immunol. 2010; 3: 69–80. 10.1038/mi.2009.109 19710636 PMC2884103

[9] Pezzulo AA, Starner TD, Scheetz TE, Traver GL, Tilley AE, Harvey BG et al The air-liquid interface and use of primary cell cultures are important to recapitulate the transcriptional profile of in vivo airway epithelia. Am J Physiol Lung Cell Mol Physiol. 2011; 300: L25–31. 10.1152/ajplung.00256.2010 20971803 PMC3023285

[10] Lopez-Souza N, Dolganov G, Dubin R, Sachs LA, Sassina L, Sporer H et al Resistance of differentiated human airway epithelium to infection by rhinovirus. Am J Physiol Lung Cell Mol Physiol. 2004; 286: L373–381. 14711802 10.1152/ajplung.00300.2003

[11] Ross AJ, Dailey LA, Brighton LE, Devlin RB. Transcriptional profiling of mucociliary differentiation in human airway epithelial cells. Am J Respir Cell Mol Biol. 2007; 37: 169–185. 17413031 10.1165/rcmb.2006-0466OC

[12] Wark PA, Johnston SL, Bucchieri F, Powell R, Puddicombe S, Laza-Stanca V, et al Asthmatic bronchial epithelial cells have a deficient innate immune response to infection with rhinovirus. J Exp Med. 2005; 201: 937–947. 15781584 10.1084/jem.20041901 PMC2213100

[13] Parsons KS, Hsu AC, Wark PA. TLR3 and MDA5 signalling, although not expression, is impaired in asthmatic epithelial cells in response to rhinovirus infection. Clin Exp Allergy. 2014; 44: 91–101. 10.1111/cea.12218 24131248

[14] Watson AM, Benton AS, Rose MC, Freishtat RJ. Cigarette smoke alters tissue inhibitor of metalloproteinase 1 and matrix metalloproteinase 9 levels in the basolateral secretions of human asthmatic bronchial epithelium in vitro. J Investig Med. 2010; 58: 725–729. 10.231/JIM.0b013e3181db874e 20305574 PMC3325291

[15] Hackett TL, Singhera GK, Shaheen F, Hayden P, Jackson GR, Hegele RG, et al Intrinsic phenotypic differences of asthmatic epithelium and its inflammatory responses to respiratory syncytial virus and air pollution. Am J Respir Cell Mol Biol. 2011; 45: 1090–1100. 10.1165/rcmb.2011-0031OC 21642587

[16] Lopez-Souza N, Favoreto S, Wong H, Ward T, Yagi S, Schnurr D, et al In vitro susceptibility to rhinovirus infection is greater for bronchial than for nasal airway epithelial cells in human subjects. J Allergy Clin Immunol. 2009; 123: 1384–1390 e1382. 10.1016/j.jaci.2009.03.010 19428098 PMC2744461

[17] Holtzman M, Patel D, Kim HJ, You Y, Zhang Y. Hypersusceptibility to respiratory viruses as a shared mechanism for asthma, chronic obstructive pulmonary disease, and cystic fibrosis. Am J Respir Cell Mol Biol. 2011; 44: 739–742. 10.1165/rcmb.2011-0120ED 21653905

[18] Chen Y, Hamati E, Lee PK, Lee WM, Wachi S, Schnurr D, et al Rhinovirus induces airway epithelial gene expression through double-stranded RNA and IFN-dependent pathways. Am J Respir Cell Mol Biol. 2006; 34: 192–203. 16210696 10.1165/rcmb.2004-0417OC PMC2644182

[19] Lennette EH. Laboratory diagnosis of viral infections: general principles. Am J Clin Pathol. 1972; 57: 737–750. 4554720 10.1093/ajcp/57.6.737

[20] Jurgeit A, Moese S, Roulin P, Dorsch A, Lotzerich M, Lee WM, et al An RNA replication-center assay for high content image-based quantifications of human rhinovirus and coxsackievirus infections. Virol J. 2010; 7: 264 10.1186/1743-422X-7-264 20937137 PMC2958916

[21] Benjamini Y, Hochberg Y. Controlling the False Discovery Rate: A Practical and Powerful Approach to Multiple Testing. Journal of the Royal Statistical Society. 1995; 57: 289–300.

[22] Ashburner M, Ball CA, Blake JA, Botstein D, Butler H, Cherry JM, et al Gene ontology: tool for the unification of biology. The Gene Ontology Consortium. Nat Genet. 2000; 25: 25–29. 10802651 10.1038/75556 PMC3037419

[23] Subramanian A, Tamayo P, Mootha VK, Mukherjee S, Ebert BL, Gillette MA, et al Gene set enrichment analysis: a knowledge-based approach for interpreting genome-wide expression profiles. Proc Natl Acad Sci U S A. 2005; 102: 15545–15550. 16199517 10.1073/pnas.0506580102 PMC1239896

[24] Woodruff PG, Boushey HA, Dolganov GM, Barker CS, Yang YH, Donnelly S, et al Genome-wide profiling identifies epithelial cell genes associated with asthma and with treatment response to corticosteroids. Proc Natl Acad Sci U S A. 2007; 104: 15858–15863. 17898169 10.1073/pnas.0707413104 PMC2000427

[25] Woodruff PG, Modrek B, Choy DF, Jia G, Abbas AR, Ellwanger A, et al T-helper type 2-driven inflammation defines major subphenotypes of asthma. Am J Respir Crit Care Med. 2009; 180: 388–395. 10.1164/rccm.200903-0392OC 19483109 PMC2742757

[26] Proud D, Turner RB, Winther B, Wiehler S, Tiesman JP, Reichling TD, et al Gene expression profiles during in vivo human rhinovirus infection: insights into the host response. Am J Respir Crit Care Med. 2008; 178: 962–968. 10.1164/rccm.200805-670OC 18658112

[27] Thomas B, Rutman A, Hirst RA, Haldar P, Wardlaw AJ, Bankart J, et al Ciliary dysfunction and ultrastructural abnormalities are features of severe asthma. J Allergy Clin Immunol. 2010; 126: 722–729 e722. 10.1016/j.jaci.2010.05.046 20673980

[28] CSGA. A genome-wide search for asthma susceptibility loci in ethnically diverse populations. The Collaborative Study on the Genetics of Asthma (CSGA). Nat Genet. 1997; 15: 389–392. 9090385 10.1038/ng0497-389

[29] Dizier MH, Besse-Schmittler C, Guilloud-Bataille M, Annesi-Maesano I, Boussaha M, Bousquet J, et al Genome screen for asthma and related phenotypes in the French EGEA study. Am J Respir Crit Care Med. 2000; 162: 1812–1818. 11069818 10.1164/ajrccm.162.5.2002113

[30] Quint JK, Donaldson GC, Goldring JJ, Baghai-Ravary R, Hurst JR, Wedzicha JA. Serum IP-10 as a biomarker of human rhinovirus infection at exacerbation of COPD. Chest. 2010; 137: 812–822. 10.1378/chest.09-1541 19837822 PMC2851557

[31] Sanchez-Cuellar S, de la Fuente H, Cruz-Adalia A, Lamana A, Cibrian D, Giron RM, et al Reduced expression of galectin-1 and galectin-9 by leucocytes in asthma patients. Clin Exp Immunol. 2012; 170: 365–374. 10.1111/j.1365-2249.2012.04665.x 23121677 PMC3518896

[32] El-Shazly A, Berger P, Girodet PO, Ousova O, Fayon M, Vernejoux JM, et al Fraktalkine produced by airway smooth muscle cells contributes to mast cell recruitment in asthma. J Immunol. 2006; 176: 1860–1868. 16424217 10.4049/jimmunol.176.3.1860

[33] Fregonese L, Swan FJ, van Schadewijk A, Dolhnikoff M, Santos MA, Daha MR, et al Expression of the anaphylatoxin receptors C3aR and C5aR is increased in fatal asthma. J Allergy Clin Immunol. 2005; 115: 1148–1154. 15940127 10.1016/j.jaci.2005.01.068

[34] Bonnelykke K, Sleiman P, Nielsen K, Kreiner-Moller E, Mercader JM, Belgrave D, et al A genome-wide association study identifies CDHR3 as a susceptibility locus for early childhood asthma with severe exacerbations. Nat Genet. 2013; 46: 51–55. 10.1038/ng.2830 24241537

[35] Di YP, Zhao J, Harper R. Cigarette smoke induces MUC5AC protein expression through the activation of Sp1. J Biol Chem. 2012; 287: 27948–27958. 10.1074/jbc.M111.334375 22700966 PMC3431669

[36] Jakiela B, Brockman-Schneider R, Amineva S, Lee WM, Gern JE. Basal cells of differentiated bronchial epithelium are more susceptible to rhinovirus infection. Am J Respir Cell Mol Biol. 2008; 38: 517–523. 18063839 10.1165/rcmb.2007-0050OC PMC2358970

[37] Tapparel C, Sobo K, Constant S, Huang S, Van Belle S, Kaiser L. Growth and characterization of different human rhinovirus C types in three-dimensional human airway epithelia reconstituted in vitro. Virology. 2014; 446: 1–8. 10.1016/j.virol.2013.06.031 24074561

[38] Jakiela B, Gielicz A, Plutecka H, Hubalewska-Mazgaj M, Mastalerz L, Bochenek G, et al Th2-type cytokine induced mucous metaplasia decreases susceptibility of human bronchial epithelium to rhinovirus infection. Am J Respir Cell Mol Biol. 2014 Aug;51(2):229–41. 10.1165/rcmb.2013-0395OC 24588727

[39] Ashraf S, Brockman-Schneider R, Bochkov YA, Pasic TR, Gern JE. Biological characteristics and propagation of human rhinovirus-C in differentiated sinus epithelial cells. Virology. 2013; 436: 143–149. 10.1016/j.virol.2012.11.002 23199420 PMC3545098

[40] Nakagome K, Bochkov YA, Ashraf S, Brockman-Schneider RA, Evans MD, Pasic TR, et al Effects of rhinovirus species on viral replication and cytokine production. J Allergy Clin Immunol. 2014; 134: 332–341. 10.1016/j.jaci.2014.01.029 24636084 PMC4119842

[41] Pritchard AL, White OJ, Burel JG, Carroll ML, Phipps S, Upham JW. Asthma is associated with multiple alterations in anti-viral innate signalling pathways. PLoS One. 2014; 9: e106501 10.1371/journal.pone.0106501 25203745 PMC4159236

[42] Wark PA, Grissell T, Davies B, See H, Gibson PG. Diversity in the bronchial epithelial cell response to infection with different rhinovirus strains. Respirology. 2009; 14: 180–186. 10.1111/j.1440-1843.2009.01480.x 19207121

[43] Contoli M, Message SD, Laza-Stanca V, Edwards MR, Wark PA, Bartlett NW, et al Role of deficient type III interferon-lambda production in asthma exacerbations. Nat Med. 2006; 12: 1023–1026. 16906156 10.1038/nm1462

[44] Sykes A, Macintyre J, Edwards MR, Del Rosario A, Haas J, Gielen V, et al Rhinovirus-induced interferon production is not deficient in well controlled asthma. Thorax. 2014; 69: 240–246. 10.1136/thoraxjnl-2012-202909 24127021

[45] Kennedy JL, Shaker M, McMeen V, Gern J, Carper H, Murphy D, et al Comparison of Viral Load in Individuals with and without Asthma during Infections with Rhinovirus. Am J Respir Crit Care Med. 2014;189: 532–539. 10.1164/rccm.201310-1767OC 24471509 PMC3977713

