# Supplementary figures and images for: Phenotypic Responses of Differentiated Asthmatic Human Airway Epithelial Cultures to Rhinovirus
Source: PLoS One. 2015 Feb 23;10(2):e0118286. doi: 10.1371/journal.pone.0118286 (PMC4338293; doi:10.1371/journal.pone.0118286)

Figure S1.

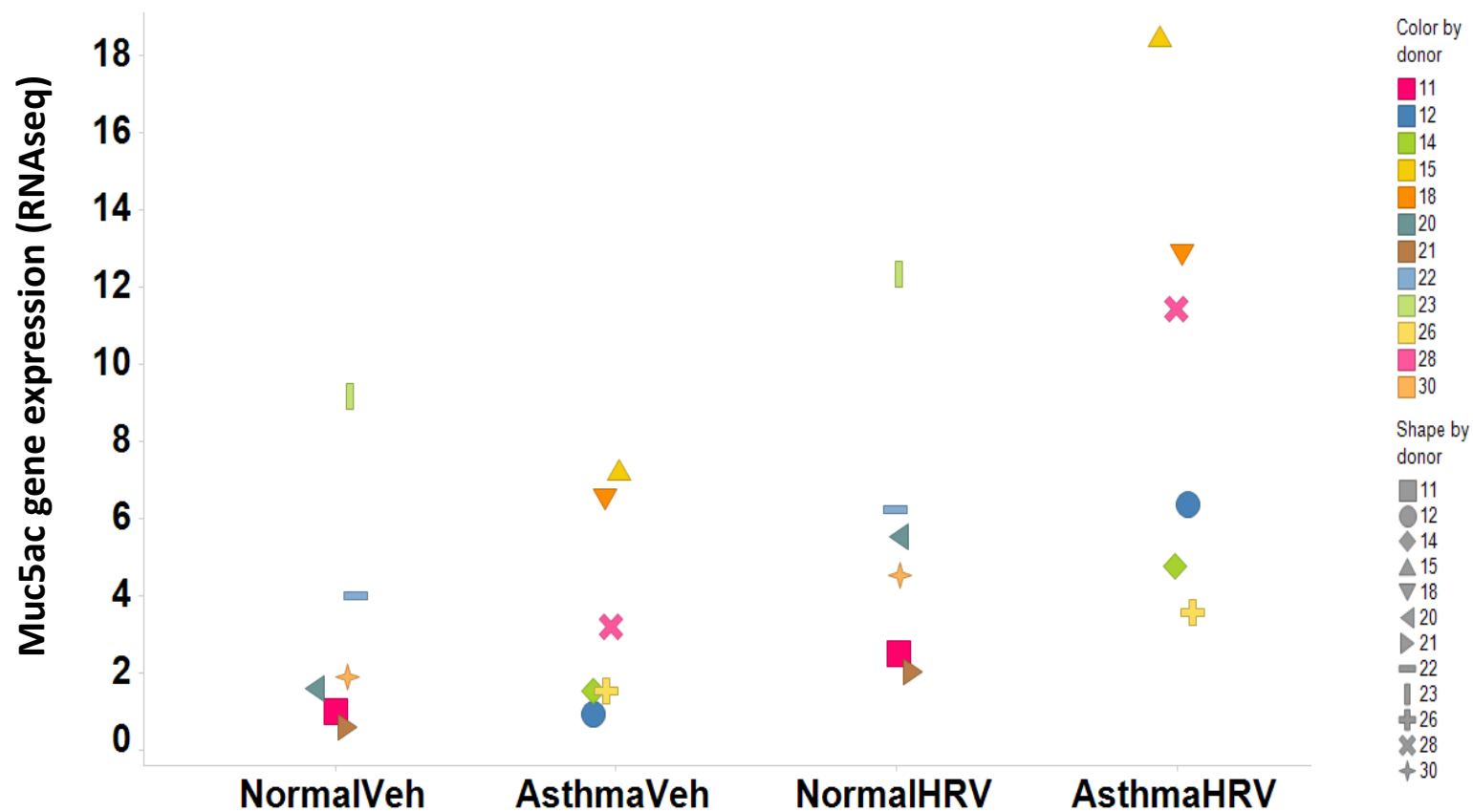

Supplement: S1 Fig — MUC5AC mRNA expression level based on RNA-seq reads was quantitated using a different annotation genome. Non-asthma donor 23 had the highest expression of MUC5AC at baseline and after HRV infection. (PDF) [file pone.0118286.s001.pdf]

Figure S2.

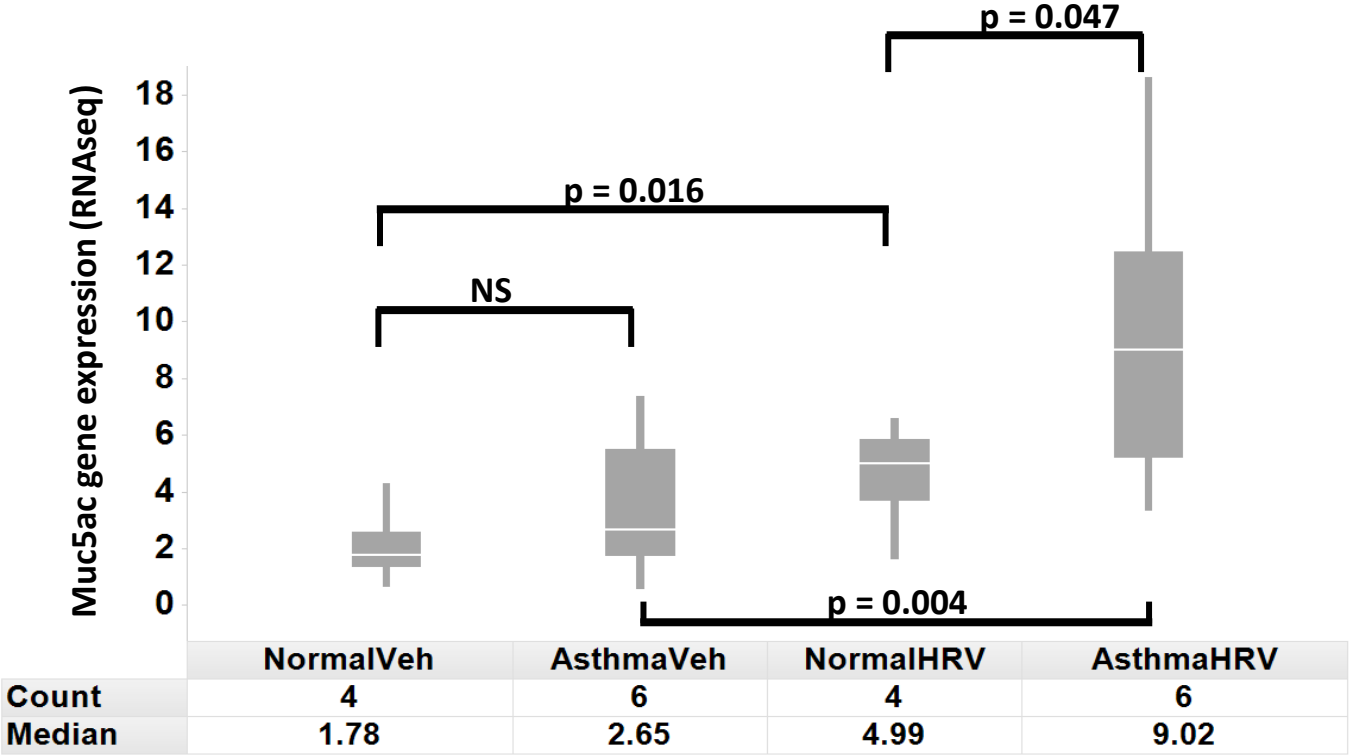

Supplement: S2 Fig — Removal of non-diseased smokers (donors 11 and 23) from the data set results in significant differences between HRV and vehicle treated tissues in both non-asthmatic and asthmatic donors, and significant differential responses to HRV between asthma and non-asthma groups as determined by ANOVA and Tukey HSD test. (PDF) [file pone.0118286.s002.pdf]
